# 

**Published:** 1835-07-01

**Authors:** 


### NOTICE TO SUBSCRIBERS.

*The Proprietors beg respectfully to inform the Subscribers, that the present Number is the last which will appear under the name of the* MEDICAL QUARTERLY REVIEW: *they have, however, the gratification to state, that, in discontinuing the publication under that title, they have taken care that the interests of the numerous friends who have so liberally patronised it, shall not in any degree be compromised, but, on the contrary, still further consulted, by the arrangements into which they have entered. Whether the* BRITISH AND FOREIGN MEDICAL REVIEW *be regarded as a continuation of the former publication, under a new designation and management, or as its successor in the path which it has left,—in either case, the Proprietors feel justified in recommending it to their Readers, as a work which they are confident will be found no less deserving their countenance and support.*

*It will be seen, by the Prospectus of the* BRITISH AND FOREIGN MEDICAL REVIEW, *which accompanies the present Number, and to which the particular attention of the Reader is requested, that the New Quarterly Review will present considerable modifications of the plan of its predecessor; but modifications which, it is believed, will be generally regarded as improvements. The principal of these are—the exclusion from the* BRITISH AND FOREIGN MEDICAL REVIEW *of all* Original Communications, *except under the form of Reviews of published works, and the extension of the department heretofore devoted to notices of* Foreign *Publications. Indeed, the latter arrangement necessarily involved the former.*

*The more extensive diffusion of the knowledge of* Foreign Medical Literature *seemed imperiously called for; and it was considered that, while numerous other channels were still open for* Original Memoirs, *the exclusion from the pages of the New Review of full Notices of Foreign Medicine and Surgery, would tend to perpetuate a most important defect in our periodical literature, which, by the new arrangement, might be satisfactorily remedied.*

*** *It was originally intended to commence the publication of the* BRITISH AND FOREIGN MEDICAL REVIEW *on the* 1st *of October, but it has been found advisable, in order to give time to complete the necessary arrangements, to postpone the appearance of the first Number to the* 1st *of January,* 1836. *This delay will have the further incidental advantage of rendering each volume complete within the limits of the same year, a circumstance attended with some convenience in the case of reference.*

---

☞ *The* INDEX *to the Volume will be delivered gratuitously on the* 1st *of October next.*

### NOTICES.

*The Review of* LOUIS *sur les Effets de la Saignée, &c. is left for the Author at our Publisher's.*

*We have received the following Books:*

*The Cyclopædia of Medicine, Parts* XXVI. *and* XXVII. (completing the Work.) ANDRAL'S *Clinique Médicale, translated by* DR. SPILLAN: *Part* I. STAPLES *on Cupping.* RIOFREY *sur la Leucorrhée.* TODD *on Diseases of the Spinal Marrow,* (from the Cycl. of Medicine.) TURNBULL *on the Medicinal Properties of the Ranunculaceæ.* JEFFRAY *on the Heart and on the Peculiarities of the Fœtus.* SPRATT'S *Obstetric Tables, 2nd Edition, Part* I. KOECKER *on Artificial Teeth, Obturators, and Palates. The American Cyclopædia of Practical Medicine and Surgery: Part* VI. Philadelphia, January, 1835.

*Any of the Back Numbers of the* Medical Quarterly Review, *from* 1 *to* 8, *to complete Sets, may be had as hitherto, of the Publisher,* J. SOUTER, 73, *St. Paul's Church-yard.*

